# ARGEOS: A New Bioinformatic Tool for Detailed Systematics Search in GEO and ArrayExpress

**DOI:** 10.3390/biology10101026

**Published:** 2021-10-11

**Authors:** Gleb E. Gavrish, Dmitry V. Chistyakov, Marina G. Sergeeva

**Affiliations:** 1 Faculty of Bioengineering and Bioinformatics, Moscow Lomonosov State University, 119234 Moscow, Russia; ggavrish@fbb.msu.ru; 2 Belozersky Institute of Physico-Chemical Biology, Lomonosov Moscow State University, 119992 Moscow, Russia; sergeeva@belozersky.msu.ru

**Keywords:** GEO, ArrayExpress, polarization, systematics search, gene expression, bioinformatic tool, transcriptome

## Abstract

**Simple Summary:**

A systematic search for datasets of transcriptome data is a hefty task. Therefore, we developed the ARGEOS web tool, which simplifies the search and selection of datasets from various public databases. In addition, the service carries out an advanced analysis of a dataset, including collecting detailed protocols, information on the number of datasets, and providing additional reference information. An example of a cell polarization study exemplifies the effectiveness of the tool.

**Abstract:**

Conduct a reanalysis of transcriptome data for studying intracellular signaling or solving other experimental problems is becoming increasingly popular. Gene expression data are archived as microarray or RNA-seq datasets mainly in two public databases: Gene Expression Omnibus (GEO) and ArrayExpress (AE). These databases were not initially intended to systematically search datasets, making it challenging to conduct a secondary study. Therefore, we have created the ARGEOS service, which has the following advantages that facilitate the search: (1) Users can simultaneously send several requests that are supposed to be used for systematic searches, and it is possible to correct the requests; (2) advanced analysis of information about the dataset is available. The service collects detailed protocols, information on the number of datasets, analyzes the availability of raw data, and provides other reference information. All this contributes to both rapid data analysis with the search for the most relevant datasets and to the systematic search with detailed analysis of the information of the datasets. The efficiency of the service is shown in the example of analyzing transcriptome data of activated (polarized) cells. We have performed a systematic search of studies of cell polarization (when cells are exposed to different immune stimuli). The web interface for ARGEOS is user-friendly and straightforward. It can be used by a person who is not familiar with database searching.

## 1. Introduction

Conducting secondary studies using transcriptome data is increasingly used as a primary method for studies of intracellular signaling [1,2], the study of molecular signatures of various diseases and conditions [3,4], the search for biomarkers of diseases [5], drug development [6], or other experimental problems. This approach makes searching for data to solve the problems of systematic analysis necessary. 

Gene expression data are archived as microarray or RNA-seq datasets mainly in two public databases: Gene Expression Omnibus (GEO; http://www.ncbi.nlm.nih.gov/geo/, 4 October 2021) and ArrayExpress (AE; https://www.ebi.ac.uk/arrayexpress/, 4 October 2021). Newly appearing local public databases have already been integrated or are planned to be integrated with one of these large databases. For example, the Japanese GEA data will be indexed by AE [7]. To date, these databases contain more than 156,000 records (datasets) and more than 4.5 million samples. Each dataset can contain one to three to several thousand samples. At the same time, it may be noted that these databases were not initially intended for searching datasets for systematic analysis, which makes it challenging to conduct secondary research.

Many services make it easier to work with the data itself (see review by Wang et al. 2019 [8]). The main directions of such programs are advanced processing of user-selected datasets with a specific focus. For example, at the moment, many tools have been developed for analyzing Array data from GEO: GEO2Enrich [9], ImaGEO [10], and GEOracle [11]. These programs allow users to analyze differentially expressed genes (DEGs) of the selected datasets by considering metadata. However, they are limited to microarray datasets. A package for R has also been developed that allows interaction with GEO data, called GEO2R [12]. Some services such as GREIN—an open-source resource for re-using GEO RNA-seq data [13]—or TACITuS—a web-based system that supports rapid query access to microarray and NGS repositories [14]—were offered to facilitate work with sequencing data.

At the same time, there is still no convenient tool for the primary selection of datasets on the topic selected by the user and, more often, the researcher has to perform this work manually, analyzing both GEO and AE. Indeed, most of the existing software solutions do not provide the ability to search and sort by research metadata, e.g., the concentration of a substance for cell experiments or the exposure time for experiments with animals, i.e., about the information stored in the research protocols. Consequently, there is a need to create a convenient tool for systematically searching datasets on a selected topic, integrating the two largest public gene expression databases, and extracting additional data from experimental protocols that characterize datasets in more detail. 

Primary dataset search services include the work of Ivliev [15], which makes it easier to find individual datasets, but this complicates systematic analysis since it does not work with experiment protocols [15]. The solution to the problem of combining duplicate datasets when searching in GEO, Array, and the Genomic Expression Archive (GEA; https://www.ddbj.nig.ac.jp/gea/, 4 October 2021) is proposed in the OAE (All Of The Gene Expression) service, which was designed to index public gene expression data [16]. OAE allows users to observe the number of entries by keywords quickly and in the form of a histogram and provides information on the number of entries, dividing them by year, type, or organism. At the same time, the proposed tool has the same drawbacks as the work of Ivliev [15]. Indeed, in order to systematically search for suitable datasets, analyzing a large pool of information is necessary, including the number of samples in the dataset, logs and the year when the dataset was published, and, very importantly, experimental protocols that include data on the concentration of stimuli, the time of their use, and other details. 

We faced the primary search for datasets for analysis when searching for transcriptome data on polarization experiments. Polarization is a complex of cellular changes that change the cellular phenotype [17,18]. Cell pro-inflammatory and anti-inflammatory phenotypes are determined, which are elicited by various stimuli, for example, lipopolysaccharide (LPS) and interleukin-4 (IL-4), respectively [17,18]. Functional phenotypes, which can be acquired by cells depending on the microenvironment or stimulation, are currently the focus of investigations into new anti-inflammatory therapeutic approaches [19,20]. Not surprisingly, there are many different transcriptome datasets in this area of research, which contain from two to thousands of samples in each and obtained from other cells, with different stimuli and different technical protocols.

Currently, there are 144,576 records in the GEO database. When searching for LPS, the GEO database produces 2178 records and 1011 records for IL-4. However, in the built-in search in GEO, filtering is possible only by organism, experiment type, or publication date. The user needs to filter the rest of the information about datasets manually. For a complex query that includes several keywords, it is necessary to formulate a complex query, combining the keywords with the operators ‘AND’ and ‘OR’. Thus, it is difficult to carry out a systematic search using standard tools. 

ARGEOS aims to combine gene expression data and make them systematically searchable in a user-friendly form. We developed the ARGEOS algorithm by indexing the publicly available gene expression databases GEO and AE. The effectiveness of our ARGEOS service is shown by the example of searching for data on studies of cell polarization when the innate immune system is exposed to LPS, IL-4, and other stimuli.

## 2. Materials and Methods

### 2.1. Implementation 

ARGEOS is written in Python 3.8 (Python Software Foundation, Wilmington, DE, USA). The search in GEO is carried out remotely by sending a request to National Center for Biotechnology Information (NCBI) E-Utilities [21]. Interaction with NCBI E-Utilities services occurs through the Entrezpy package [22]. The package provides a communication delay and fulfills other NCBI recommendations. The search in AE is performed by parsing the XML output, and the query is formed so that only the unique AE datasets are returned.

### 2.2. Obtaining GEO Data 

Data analysis of GEO datasets is performed by analyzing an XML file located in the Miniml section on the NCBI GEO FTP server. 

### 2.3. Obtaining AE Data 

Parsing the records in the database is performed by querying the EBI AE service (www.ebi.ac.uk/arrayexpress/, 4 October 2021). For each record found, an XML file is formed, which the program reads, distributes the information into variables, and prints in the final output. Protocol data are obtained separately and upon request by ID.

### 2.4. Data Analysis 

GEO: The following information is collected from the block with information about the dataset: organism (organisms), number of samples (samples), experiment type (types), platform ID, dataset name, upload date, summary, and general design of the experiment. The ID (from the PubMed database) of the reference article is also collected. The following information is collected from the block of samples: cell type, treatment protocol, growth protocol, type of analyzed molecules, isolation protocol, and the characteristics field, which includes all other parameters of the sample. Sample information is filtered to remove duplicates. The user then receives one record entry. If the sample information is different, i.e., unique for each sample, then the user receives multiple protocols. For example, if the treatment protocol is the same for all samples, the user will receive it only once. However, if the authors provided a unique treatment protocol for each sample, the user will receive them all. AE: For each record found, an XML file is formed, which the program reads, distributes the information into variables, and prints in the final output. Thus, there is a union of two databases and the end-user receives the same information in the same columns, which conveys the appearance of data homogeneity. This is intended to facilitate the operations of filtering data and reading data for manual sorting or classification. 

### 2.5. Analyzing PubMed Data

For each dataset from GEO and AE, if a reference article is specified, the program receives a PubMed ID or DOI, through which it searches PubMed by sending a request to NCBI E-Utilities [15]. The final table contains information about the title of the article, DOI, the name of the journal, and the impact factor calculated based on the name of the journal.

### 2.6. Data Export

After writing information about datasets into variables and bringing the information into the same format, the information is written to “EXPORT TABLE 2” in the following order.

Accession—record ID (GEO or AE); Organism—an organism (or organisms) specified by the authors; Samples—number of samples; Type—the type of experiment; Platform—the platform (or platforms) on which the raw data is received. Always indicated GEO, less commonly found in AE; Title—the name of the dataset; Year—the date of the first publication of the dataset; Summary—a summary dataset and a summary of the study provided by the authors; Link—link to the dataset (in GEO or AE); next is the block of reference articles. All articles indicated by the authors as related to this dataset are listed in the “All references” block. Among them, the article published in the journal with the highest impact factor stands out. Information about this article is listed in separate columns: Paper_title—the title of the reference article; Journal—the journal in which the reference article was published; Impact factor—the impact factor of the magazine; DOI or PubMed—link to the article; All references—information on all articles (titles, magazines, and links); Type of molecule—the type of molecule analyzed in the study (for example, “total RNA” or “Genomic DNA”); BioProjectlink (NCBI)—Link to the BioProject page on the NCBI website; BioProjectlink (EBI)—Link to the BioProject page on the EBI website (EMBL); SRA—link to SRA (relevant for RNA-seq experiments); All protocols—the column contains all the textual information provided by the authors of the dataset. These can be protocols, sample characteristics, etc. If the number of characters exceeds the limit allowed for most software, then the information is split into several cells (in adjacent columns) in order to correctly read the file.

### 2.7. Source Code Availability 

A stand-alone version of the program is available for download on Github (https://github.com/gleb-gavrish/ARGEOS, 4 October 2021). The program is available in the .py extension, which allows anyone to access the source code.

## 3. Results

### 3.1. ARGEOS Work Algorithm 

**Figure 1:**
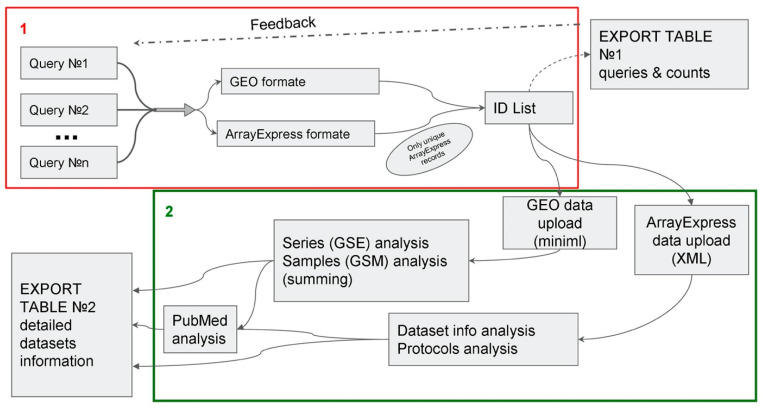
Diagram of the ARGEOS web tool. There are two blocks in the ARGEOS scheme: (**1**) search; (**2**) collection of information. When performing a search, ARGEOS accepts search queries as inputs and searches the GEO and AE databases. Based on the results of the first block, a table is formed with the number of finds, as well as a list of IDs of all finds (without repetitions). The second block collects all information on each find. After bringing the information into a unified form, a final table is formed, with all the findings on request enriched with detailed information. For an explanation of the individual operations, see the main text.

For the task of systematic analysis of transcriptome data, we proposed the following scheme of the program (Figure 1). The circuit consists of two main blocks. 

Block #1 Search: In this block, the user makes requests and enters the keywords. Next, the corresponding datasets are selected and filtered from the GEO and AE databases (they are compared and duplicates removed), and a list is created with the ID of all found datasets. It is important to note that the ID sheet contains data on both microarrays and RNA-seqs, methylation, and all other experiments presented in these databases. At the same time, a table (“EXPORT TABLE #1”) is saved with information about the request and the number of datasets corresponding to this request. This allows the user to adjust the search at this stage. Table S1 shows an example of the algorithm output for the queries for “polarization”, which includes 23 unique queries. 

Block #2 Gathering information: Since GEO and AE have different recording formats for their databases, our program combines the data from these databases into a single form. In GEO, we receive detailed information from a file in the Miniml format (Figure 1). In parallel, we analyze information related to series (with an ID in the format GSEnnn) and samples (with an ID in the format GSMnnn). In the series section, basic information is recorded, such as the type of experiment, the number of samples, and summary. The sample section stores information about research protocols and various experiment parameters, such as cell type or incubation time. AE provides information in XML format (Figure 1). A file of this format contains all the basic information except for the research protocols. Only IDs for the corresponding protocols are indicated. By using these IDs, the texts of the protocols are separately collected, which are located in separate XML files. 

The last stage of the analysis is collecting information about the PubMed article (Figure 1). In collecting basic information in the previous steps, article IDs are collected in the PubMed database. By using a call to the PubMed database, information is then collected about title of publications, year of publication of articles, impact factors of the journals, and DOIs. This ensures the same article information format for entries from GEO and AE. It is important to note that the program breaks down datasets with several organisms or experiment types into several rows (e.g., samples obtained on several organisms or in the course of experiments of different types). This makes it convenient to filter records even if there are multiple values in the original field. This feature is enabled by default but can also be disabled with the appropriate flag. 

After block 2 is completed, “EXPORT TABLE #2” is created. The table includes the following data: Accession—ID of a dataset from GEO or AE; Organism—an organism or organisms (if several); Samples—number of samples; Type—the type or types of the experiment (chips, pH-sec, etc.); Platform—ID of the platform on which the experiment was carried out; Title—the name of the dataset; Year—the year the dataset was published; Summary—abstract of the dataset specified by the authors; Link—link to GEO or AE; Paper_title—the title of the article; Journal—the journal in which the article is published; Impact factor 2018—impact factor; DOI or PubMed ID—link to the article; All references—information on all articles (if there are several); Type of molecule—a type of detected molecules; BioProject link (NCBI)—link to BioProject (page in the NCBI database); BioProject link (EBI)—link to BioProject (page in EBI database); SRA—link to record in SRA; All protocols—all protocols specified by the authors. An example of a complete table with the output of the algorithm for queries “polarization” is given in Table S2. 

### 3.2. Graphical Web Interface 

In order to simplify the user’s work with tools, we have developed a web interface for the program, which is available at www.ar-geos.org, 4 October 2021.

**Figure 2:**
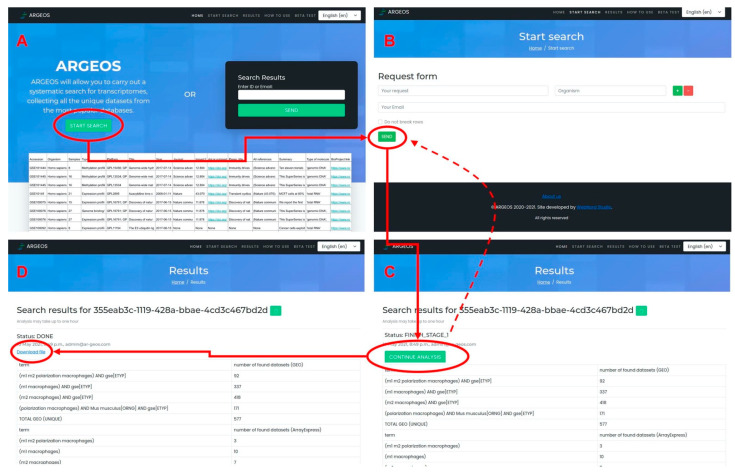
ARGEOS web interface: workflow analysis. (**A**) shows the home page interface. (**B**,**C**) show the pages for interacting with the service, and (**D**) shows the completed analysis page with a link to the final file.

The action of the ARGEOS tool was implemented as a user-friendly web interface (Figure 2). The user steps are simple: follow the link to the interface (A), click Run, go to the search page (B), enter a query or several queries into the fields, click Run and the program starts the search (>minutes), and EXPORT TABLE #1 (C) appears. The user then clicks on the Run button and the analysis continues with the formation of “EXPORT TABLE 2” (D). If the query is large, the formation of “EXPORT TABLE 2” can take up to an hour. After that, the table is available for download. A tutorial that shows how to use the ARGEOS web interface is available on YouTube (https://www.youtube.com/watch?v=9V3YWkVejac, 4 October 2021).

### 3.3. Creation of RNA-seq Datasets Using ARGEOS to Study the Phenomenon of Changes in Cellular Phenotype 

We applied the developed approach to the collection of datasets dedicated to cellular polarization. Polarization is a complex set of cellular changes that result in a change in the cellular phenotype. This effect was first shown for macrophages [23,24]. Currently, similar polarization effects have been shown for cells of the central nervous system, e.g., microglia [25] and astrocytes [26,27,28]. Polarization has been linked to the development of many diseases [29]. In model experiments, LPSs are used to change the cellular phenotype towards an inflammatory response and IL-4 towards an anti-inflammatory response [29]. 

We aimed to analyze the work on the study of polarization at the transcriptome level. The general scheme of information retrieval is the same as recommended for systematic reviews [30,31]. According to Figure 1, formed queries made it possible to obtain EXPORT TABLE #1 (Supplementary Table S1) and EXPORT TABLE #2 (Supplementary Table S2). Thus, a search with the ARGEOS tool yielded 1691 records. 

For secondary analysis of transcriptomes, we chose RNA-seq experiments. Since the peculiarities of the GEO database are that individual samples are recorded as a new dataset when combined with a new ID (the so-called “SuperSeries”), we also filtered them. This resulted in a table with 472 datasets (Supplementary Table S3). 

**Figure 3:**
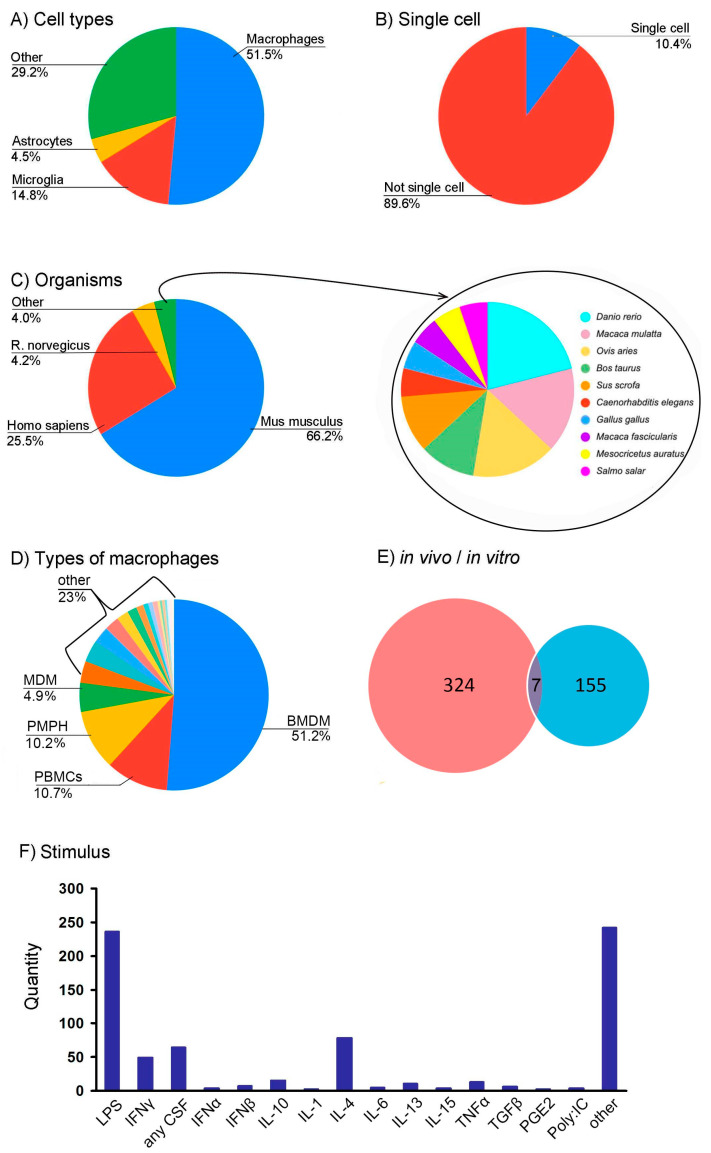
Analysis of the obtained local database for queries related to polarization. Distribution of datasets (**A**) by the type of cells on which the studies were carried out; (**B**) by data on individual cells or populations; (**C**) by the organism from which the cells were obtained; (**D**) by methods for isolating macrophages (their subtypes); (**E**) by whether stimulation took place in vivo or in vitro; and (**F**) by stimuli used to change the cellular phenotype. Abbreviations: BMDM—Bone-marrow-derived macrophages; PBMCs—peripheral blood monocytes cells; PMPH—peritoneal macrophages; MDM—monocytes derived macrophages; LPS—lipopolysaccharide; IFN—interferon; CSF- colony-stimulating factor; IL—interleukin; TNFα—tumor necrosis factor alpha; TGFβ—transforming growth factor beta; PGE2—prostaglandin E2; Poly:IC—polyinosinic-polycytidylic acid.

Next, we supplemented the table with a markup made by using manual binary classification (0 if there is no feature; 1 if there is a feature). This allowed us to mark the following features of interest: cell types (macrophages, microglia, astrocytes, and others), stimuli (LPS, IL-4, INFγ, TNFα, etc.), and type of experiment (in vitro, in vivo, and single-cell). Table S3 includes all RNA-seq datasets devoted to polarization and contains information about the experimental parameters, which allows for targeted comparison of specific transcriptomes to obtain adequate valid data. Thus, using the ARGEOS program and the additional processing carried out, it was possible to create a local database to study polarization processes. Data on the ratio of dataset parameters are shown in Figure 3.

Although polarization has been described for different cell types [32], the attention of researchers is focused on macrophages (51.5%) (Figure 3A). Some studies (10.4%) were carried out on individual cells (Figure 3B). In total, studies were carried out on 13 organisms, most often Mus musculus (66.2%) and then Homo sapiens (25.5%) (Figure 3C). When working with cell cultures for planning studies and analyzing results, it is informative to distinguish between obtaining cells. In total, we identified 21 different types of macrophages. Most studies were carried out on bone marrow monocyte-derived macrophages (BMDM; 51.2%) (Figure 3D). Datasets numbering 317 refer to in vitro experiments, 148 to in vivo, and both types of data are available for seven datasets (Figure 3E). We also analyzed the stimuli used to obtain different polarization studies’ phenotypes (Figure 3F). The local database contains the distribution for all possible incentives, i.e., substances with which they acted on cells or organisms by injection. Other influences (such as hypoxia) and various miRNAs and siRNAs belong to the other field (Figure 3F). 

Thus, the analysis of the local database data built with ARGEOS allows users to receive a primary idea of the state of research in this area of interest. 

## 4. Discussion

Transcriptome analysis technologies are important systems biology methods for investigations. The two most commonly used techniques for transcriptome analysis (microarray technology and next-generation sequencing) generate a large amount of data. Most existing tools dealt with the storage and analysis of initial data. Recently, the analysis of transcriptomic data has started to be used as a preliminary search before using usual molecular biology methods such as real-time PCR or overall planning of the experimental design of research. Such new tasks raise the problem of searching transcriptomic data.

There are currently two primary public databases, GEO (http://www.ncbi.nlm.nih.gov/geo/, 4 October 2021) and AE (https://www.ebi.ac.uk/arrayexpress/, 4 October 2021). The AE database is currently being migrated to the BioStudies database [33]. The complete transition is expected in 2021, after which AE will be completely phased out. However, old and new data will be available through the BioStudies database, and the presence of two large databases will remain. To date, GEO and AE contain more than 156,000 records, which contain information on more than 4.5 million samples. This number will increase exponentially. Therefore, the ARGEOS tool is a convenient search tool, as it allows users to consider different formats for placing source data in databases and provides much more additional information for systematic analysis. 

The ARGEOS service has the following advantages:

1. Users can send several queries simultaneously, which are supposed to be used for systematic searches.
2. It is possible to analyze the number of finds for each query (this allows the user to adjust queries in advanced searches).
3. Advanced analysis of information about the dataset is available.

The service collects detailed logs, information on the number of datasets, analyzes the availability of raw data, and provides other reference information including link and journal impact factors. All this contributes to both rapid data analysis with the search for the most relevant datasets and detailed search with detailed analysis of the information of the datasets. This is the first tool with these capabilities, which surpasses the previously proposed tools of Ivliev [15] or Bono [16].

We considered that researchers of different specialties could search transcriptome data. Therefore, we made a user-friendly and straightforward web interface for ARGEOS which can be used by biologists who are not familiar with database searching.

We have previously used transcriptomic data analysis to study gliomas [34], cell cilia [35], or systematic mining of gene co-expression networks in cancer [36]. In previous years, there was no problem with data searching, and special tools were not required. There were few data, or the searches did not need to retrieve protocol details. The problem arose when investigating the molecular mechanisms of inflammatory processes on the cellular level. The inflammatory response is part of the innate immune response and accompanies many diseases such as infection, chronic inflammation, metabolic diseases, neurodegeneration, mental diseases, and cancer [37]. Polarization is a small part of modern science, but large amounts of data have accumulated in transcriptome storage databases. As it was impossible to process these data manually, we created a special ARGEOS tool for solving the problems of systematic analysis of transcriptomic data.

We also demonstrated the possibilities of using the ARGEOS tool in the example of searching and conducting primary analysis of transcriptome data in the field of studying changes in the phenotype of cells under the action of stimuli associated with activation of the innate immune system. Using the ARGEOS tool allows users to create a local database on the selected topic. However, if the local database is large enough (for example, more than 100 records), its analysis requires different approaches. Since our local database contained more than 400 records, we described the steps we took for the analysis, including the manual data processing algorithm we used. This is performed to facilitate compiling and processing local databases for any study of this type. Creating local databases appears to be a practical solution to the problem of systematic analysis of transcriptome data. Such local databases, being in the public domain, can be an additional effective source of information. For example, the local database on the polarization problem will allow researchers to reanalyze data in future, conveniently identify standing markers of polarization coordinates, and identify the critical nodes of polarization metabolism. A new time has come and, for the first time, we offer a ready-made tool and pipeline for the systematic search of datasets.

The example with polarization provided in the work allowed us to perform the following: (1) isolate macrophages as cells for studying the polarization process; (2) postpone research on single cells (due to their weak representation); (3) choose a mouse as the most studied organism; (4) choose a source of macrophages isolation (BMDM); (5) choose a couple of stimuli LPS-IL-4; (6) choose incubation time with the stimulus; and (7) select datasets for analysis. It would take a long time to carry out this analysis without using ARGEOS. Thus, our tool not only simplifies large-scale data collection but also facilitates their selection for analysis and design of further experimental work.

## 5. Conclusions

The accumulation of data in open transcriptome databases and the intensive use of transcriptome analysis for secondary research or the design of experiments have set the task of developing a special service. ARGEOS is a bioinformatic tool for a deep scan of transcriptomic datasets that contributes to systematic search with detailed analysis on the information of the datasets.

## Data Availability

Data are contained within the article or supplementary material.

## Supplementary Materials

The following are available online at https://www.mdpi.com/article/10.3390/biology10101026/s1, Table S1: Requests, Table S2: Program output, Table S3: Polarization database.
